# Ibuprofen Overuse Leading to Life-threatening Hypokalemia Associated with Renal Tubular Acidosis in Two Patients

**DOI:** 10.7759/cureus.6404

**Published:** 2019-12-17

**Authors:** Nikhila Thammineni, Pradeep R Kathi, Aditi Sharma, Areeba Jawed

**Affiliations:** 1 Internal Medicine, University of New Mexico, Albuquerque, USA; 2 Internal Medicine / Gastroenterology, University of Arizona, Tucson, USA; 3 Internal Medicine, Wayne State University School of Medicine, Detroit, USA; 4 Internal Medicine / Nephrology, Wayne State University, Detroit, USA

**Keywords:** ibuprofen, nsaid, renal tubular acidosis, hypokalemia

## Abstract

Ibuprofen is a commonly used medication in the United States and is used both by prescription and over the counter, while hypokalemia is a life-threatening condition caused by various etiologies, one of which is the side effect of medications. Ibuprofen is well-known for its various nephrotoxic side effects, including hyperkalemia as a common electrolyte abnormality, however, renal tubular acidosis leading to hypokalemia with the use of ibuprofen has been reported rarely. We present here two cases of life-threatening hypokalemia due to over-the-counter use of large doses of ibuprofen and describe its management.

## Introduction

Ibuprofen-induced hypokalemia was first described by Gaul et al. [1] in 1999, and since then, there have been a total of 16 cases reported per a PubMed search [1-13]. Interestingly, most cases have been reported from Australia, UK, with one case from Mexico and one from the USA. It is most likely due to the availability of a combination of ibuprofen and codeine as an over-the-counter (OTC) medication in Australia and the UK [2]. Patients can present with potassium levels as low as 0.9 mMol/L [3], and due to the OTC availability of ibuprofen, information about its use may not be readily available in the medical records. In both our patients, a detailed medication history provided us information about OTC ibuprofen use. The cases discussed here highlight the uncommon complication of widely available ibuprofen and the importance of obtaining OTC medication history while evaluating a case of hypokalemia.

## Case presentation

Patient 1

**Figure 1:**
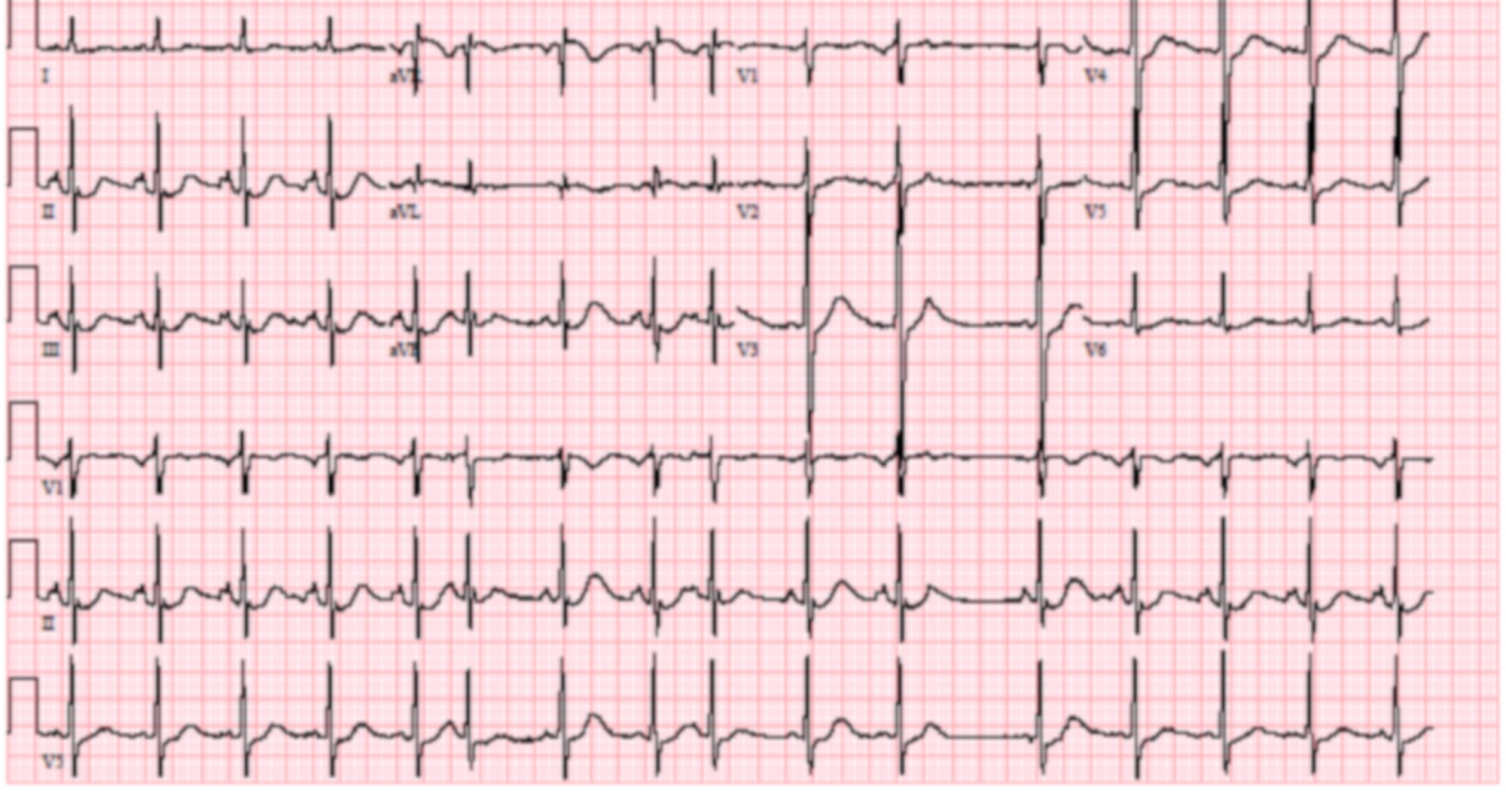
Electrocardiogram (EKG) of patient 1 showing mild QT prolongation

A 52-year-old African American male with a medical history of uncontrolled hypertension, chronic obstructive pulmonary disease (COPD), current smoker with a 20 pack-year smoking history, and arthritis was sent to the emergency department (ED) by his primary care provider, with severe hypokalemia on routine lab work. On a review of systems, he endorsed chronic right shoulder pain, back pain, loss of weight about 15 pounds in the last two months, loss of appetite, and difficulty with walking up and down the stairs for the past few days before presentation. On presentation to the ED, vitals were within normal limits. Examination showed normal strength in both the upper and lower extremities with no focal weakness. Initial workup showed significant hypokalemia of 1.9 mMol/L, bicarbonate 17 mMol/L, anion gap (AG) 16 mMol/L, and albumin 3.6 gm/dL (Table 1). The patient denied recent vomiting, diarrhea, an increase in urine output, intake of any food/herbal supplements, chewing tobacco, and the use of diuretics. He drank alcohol occasionally and urine drug screen was positive for marijuana and opiates. Electrocardiogram (EKG) showed mild QT prolongation (Figure 1). He was treated with intravenous (IV) potassium chloride (KCL) and admitted to the intensive care unit (ICU). On further interview, he reported having been taking ibuprofen for the past three months. He started with five tablets/day and increased it to 35 tablets/day to achieve adequate pain control. Other medications included acetaminophen and tramadol.

Meanwhile, a chest X-ray obtained in the ED showed a left upper lobe mass, and a subsequent CT chest showed a 7 cm left upper lobe mass, a 2 cm right upper lobe mass, and a 7 cm paraspinal mass extending into the thoracic spinal canal. Neurosurgery was consulted for surgical evaluation and he was started on dexamethasone 4 mg every six hours on day two of admission.

On day two, potassium was 2.0 mMol/L after repleting with 140 milliequivalents (mEq) of KCL on day one. The urine potassium to creatinine ratio was 48.9 mEq/gm, indicating renal potassium loss. A paraneoplastic syndrome causing hypokalemia was suspected as a possible etiology but adrenocorticotropic hormone (ACTH) was found to be low (<5 pg/ml). Hyperaldosteronism was ruled out, as the serum aldosterone was < 1.0 ng/dL, and he remained normotensive through the hospital course. Urine anion gap was found to be negative, as detailed in Table 1 and urine pH was 7, pointing toward proximal renal tubular acidosis (RTA). Uric acid was within the normal limits (3.5 mg/dL).

**Table 1:** Serum and urine biochemistries of patients 1 and 2 N/A: Not applicable

Serum lab values at the time of admission	Patient 1	Patient 2		Reference range
Sodium	139		139	136-145 mMol/L
Potassium	1.9		2.0	3.5-5.1 mMol/L
Chloride	106		106	98-107 mMol/L
Bicarbonate	17		17	21-31 mMol/L
Anion gap	16		16	5-15 mMol/L
Blood urea nitrogen	12		85	7-25 mg/dL
Creatinine	1.0		3.9	0.7-1.3 mg/dL
Calcium	9.2		9.0	8.6-10.8 mg/dL
Magnesium	2.8		1.8	1.9-2.7 mg/dL
Phosphorous	3.3		4.9	2.5-4.5 mg/dL
Albumin	3.6		2.1	3.5-5.7 gm/dL
Glucose	134		116	80-130 mg/dL
Lactic acid	0.7		0.9	0.4-2.0 mMol/L
Delta-delta gap	0.57		0.57	
Creatine kinase (day 4)	339		N/A	30-223 Units/Liter
Osmolality (day 4)	286		N/A	275-305 mOsm/kg
Urine	On Day 2		On Day 4	
p^H^	6.0		6.0	5.0-8.5
Sodium mMol/L	81		17	N/A
Potassium mMol/L	30.7		23.7	N/A
Chloride mMol/L	127		28	N/A
Anion gap	-16		12.7	N/A
Creatinine mg/dL	62.7		114.6	40-278
Osmolality mOsm/kg	382 (day 4)		N/A	50-1400 mOsm/kg

**Figure 2:**
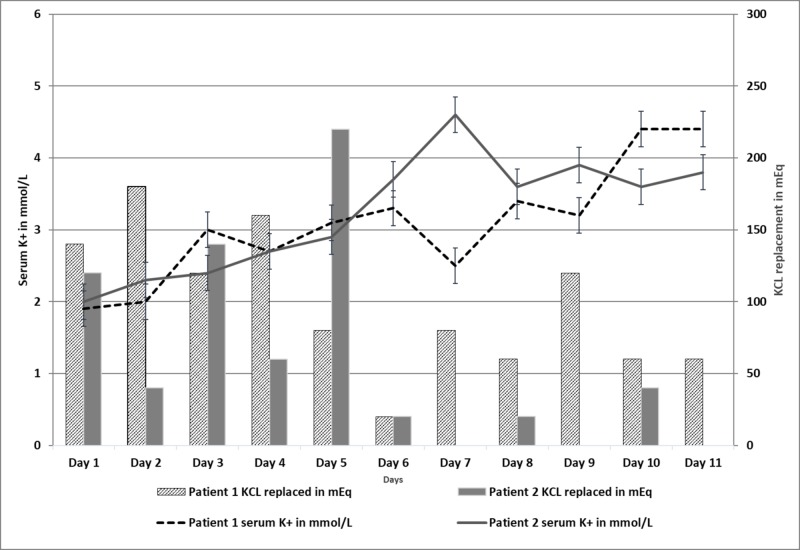
Both patients’ serum potassium on the primary Y-axis, amount of potassium chloride replaced on the secondary Y-axis, and the duration of hospitalization on the X-axis K+: potassium, KCL: potassium chloride

The patient continued to have hypokalemia despite daily replacement, as shown in Figure 2. Further workup, including autoimmune workup, serum protein electrophoresis (SPEP), and urine protein electrophoresis (UPEP) to evaluate for light chains was negative. He eventually underwent laminectomy and resection of part of the paraspinal mass on day four, and the biopsy showed poorly differentiated carcinoma with clear cell features.

After ruling out the other possible etiologies, a diagnosis of Ibuprofen-induced hypokalemia and proximal RTA was reached. The patient was aggressively treated with intravenous fluids (IVF) and he received a total of 1080 mEq of KCL replacement over 11 days of hospitalization (Figure 2) along with discontinuation of ibuprofen. The patient was subsequently discharged on day 11 with a potassium of 4.4 mMol/L.

Patient 2

**Figure 3:**
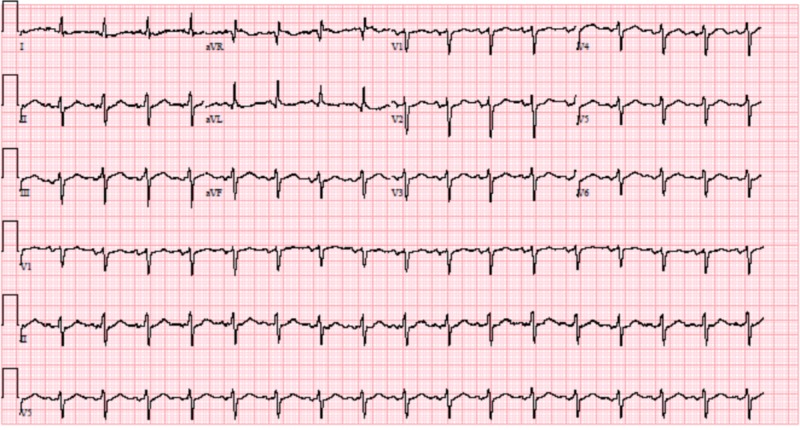
Electrocardiogram (EKG) of patient 2 showing normal sinus rhythm

A 63-year-old African American female with a history of chronic venous stasis ulcers, an unknown history of renal disease presented to the ED with complaints of epigastric pain, nausea, and vomiting for 10 days. On presentation, vitals were within normal limits and physical examination was unremarkable except for chronic ulcers on bilateral lower extremities. Her initial lab results were significant for a potassium of 2.0 mMol/L, bicarbonate of 17 mMol/L, AG of 16 mMol/L, blood urea nitrogen (BUN) of 85 mg/dL, and creatinine of 3.9 mg/dL. The rest of the labs are outlined in Table 1. Electrocardiogram (EKG) showed normal sinus rhythm (Figure 3). The patient was given 120 mEq of KCL, started on IVF, and admitted for the management of hypokalemia, renal impairment, and abdominal pain workup. On further questioning, she reported taking ibuprofen 800 mg on an average of 10-12 tablets/day for the past 10 months to help with the pain from chronic venous stasis ulcers. She denied taking any other medications, having diarrhea, and episodes of hypokalemia in the past.

Potassium on day two of admission remained low at 2.3 mMol/L and even after 180 mEq of KCL was repleted, it continued to be low at 2.7 mMol/L on day four. Meanwhile, the urine potassium/creatinine ratio was obtained on day four, and it was 20.6 mEq/gm, indicating urine loss of potassium. The patient remained normotensive, and the serum aldosterone was 2 ng/dL, ruling out hyperaldosteronism. The urine anion gap was found to be positive 12.7 and urine pH was 6 pointing toward the distal renal tubular acidosis (dRTA).

After ruling out the other possible etiologies, a diagnosis of ibuprofen-induced hypokalemia and distal RTA was reached. She was aggressively treated with fluid and electrolyte replacement, as shown in Figure 2, along with the cessation of ibuprofen. The patient was discharged on day 11 after persistent normal potassium levels were observed and serum creatinine improved to 2.2 mMol/L with a plan to follow up as an outpatient. During hospitalization, she received a total of 640 mEq of KCL replacement and her potassium was 3.8 mMol/L on the day of discharge.

## Discussion

Ibuprofen, a nonsteroidal anti-inflammatory drug (NSAID) is among the very commonly used medications with over 21.3 million prescriptions in 2016 [14], and the data about OTC use is less precise. Ibuprofen is associated with acute renal failure, chronic kidney disease, nephrotic syndrome, and interstitial nephritis but little is known about the much rarer RTA [4]. Ibuprofen-induced RTA has been hypothesized to be secondary to the inhibition of carbonic anhydrase inhibitor (CA) II, which is widely distributed throughout the nephron [3]. It has been shown to cause both proximal and distal RTA, as seen in the previously documented cases [4-5]. A PubMed search showed only 16 cases of ibuprofen-induced hypokalemia and RTA as mentioned in Table 2 [1-13]. The majority of the patients were females (10) and the mean age was 41 years, with age ranging from 28-72 years [1-13].

**Table 2:** Previously published cases of NSAID-induced hypokalemia NSAID: Nonsteroidal anti-inflammatory drug, OTC: Over the counter, RTA: Renal tubular acidosis, N/A: Not available

Author and year	Country	Age, Sex	Dose and duration of Ibuprofen	Prescription/OTC use?	Presenting potassium	Type of RTA
Patil S et al [6] (2018)	USA	48, Female	4 g/day for 3 months	N/A	1.2 mMol/L	Distal RTA
Jonathan S. Chávez-Iñiguez et al [3]	Mexico	42, Male	3.2 g/day for 5 months	OTC	0.9 mMol/L	Distal RTA
Bichard et al [2] (2017)	Australia	36, Female	8 g/day for 5 weeks in combination with codeine	OTC	2.3 mMol/L	Distal RTA
Dang et al [5] (2016)	Australia	32, Male	12 g/day for 14 years in combination with codeine	OTC	2 mMol/L	Proximal RTA
Salter et al [7] (2013)	Australia	38, Female	1.2-2 g/day for 5 weeks in combination with paracetamol, codeine phosphate, and doxylamine succinate)	OTC	2.1 mMol/L	Distal RTA
Blackstock et al [8] (2012)c	UK	38, Female	4-8 g/day for few weeks in combination with codeine	N/A	1.9 mMol/L	RTA
Ng JL et al [4] (2011)	Australia	32, Female	0.6-4 g/day for a prolonged period in combination with codeine	N/A	1 mMol/L	Distal RTA
Ng JL et al [4] (2011)	Australia	37, Male	4.8 g/day for several years in combination with codeine	N/A	2 mMol/L	Distal RTA
Ng JL et al [4] (2011)	Australia	45, Female	9.6-14.4 g/day for several months	N/A	2 mMol/L	Distal RTA
Ng JL et al [4] (2011)	Australia	40, Male	1.4-2.0 g/day for 3 months	N/A	1 mMol/L	Proximal RTA
Ernest et al [9] (2010)	Australia	39, Male	14.4 g/day for 3 days	N/A	1.8 mMol/L	
Ter et al [10] (2008)	UK	36, Female	1.6 g/day for 3 months in combination with codeine	N/A	1.7 mMol/L	Distal RTA (on 2^nd^ admission)
Lambert et al [11] (2005)	UK	45, Female	28 gm/day for an unknown duration in combination with codeine	OTC	2.6 mMol/L	Not documented
Dyer et al [12] (2004)	UK	49, Male	6 gm/day for 3 days in combination with codeine	N/A	2 mMol/L	Not available
Chetty et al [13] (2003)	UK	28, Female	8-12 g/day intermittently for 2-3 years in combination with codeine	OTC	1.4 mMol/L	Proximal RTA
Gaul et al [1] (1999)	Germany	72, Female	4.8 g/day for an unknown duration	N/A	1.4 mMol/L	Proximal RTA

The most prominent abnormalities seen in our patients are hypokalemia, metabolic acidosis likely secondary to proximal RTA, with a negative urinary anion gap in patient 1, and distal RTA, with a positive urinary anion gap in patient 2, and alkaline urine pH in the setting of ibuprofen overuse. After ruling out other possible etiologies for RTA, a diagnosis of ibuprofen-induced RTA, leading to hypokalemia, was made in both the patients.

## Conclusions

The cases described here highlight the importance of inquiring about OTC medication history and considering ibuprofen as one of the differentials in patients with a combination of refractory hypokalemia and RTA. They also stress the need to further educate patients regarding the multiple nephrotoxic effects of excessive NSAID use, including RTAs, and emphasize the importance of limiting NSAID use.

## References

[1] Gaul C, Heckmann J, Druschky A, Schöcklmann H, Neundörfer B, Erbguth F (1999). Renal tubular acidosis with severe hypokalemic tetraparesis after ibuprofen intake [Article in German]. Deut Med Wochenschr.

[2] Bichard L, Toh D (2017). Ibuprofen-induced distal (type 1) renal tubular acidosis and hypokalaemia: the dangers of ibuprofen-codeine combination over-the-counter preparations. Intern Med J.

[3] Chávez-Iñiguez JS, Espinosa-García F, Pacheco-Plascencia A, Andrade-Sierra J, Rubio-Reynoso R, García-García R (2018). The case | severe hypokalemia and metabolic acidosis. Kidney Int.

[4] Ng JL, Morgan DJR, Loh NK, Gan SK, Coleman PL, Ong GS, Prentice D (2011). Life-threatening hypokalaemia associated with ibuprofen-induced renal tubular acidosis. Med J Aust.

[5] Dang MH, Wu S, Sia C (2016). Ibuprofen-induced renal tubular acidosis-a rare cause of rhabdomyolysis: a case report. Oxford Med Case Reports.

[6] Patil S, Subramany S, Patil S, Gurram P, Singh M, Krause M (2018). Ibuprofen abuse—a case of rhabdomyolysis, hypokalemia, and hypophosphatemia with drug-induced mixed renal tubular acidosis. Kidney Int Reports.

[7] Salter MD (2019). Ibuprofen-induced hypokalemia and distal renal tubular acidosis: a patient’s perceptions of over-the-counter medications and their adverse effects. Case Rep Crit Care.

[8] Blackstock MJ, Lee A (2019). Hypokalaemia and renal tubular acidosis due to abuse of Nurofen Plus. Case Reports Crit Care.

[9] Ernest D, Chia M, Corallo C (2010). Profound hypokalaemia due to Nurofen Plus and Red Bull misuse. Crit Care Resusc.

[10] Ter A, Salha R, Vadamalai V, Soper C (2008). Ibuprofen codeine combination precipitating severe hypokalaemia in a patient with pre-existing type 1 renal tubular acidosis. NDT Plus.

[11] Lambert AP, Close C (2005). Life-threatening hypokalaemia from abuse of Nurofen Plus. J R Soc Med.

[12] Dyer BT, Martin JL, Mitchell JL, Sauven NC, Gazzard B (2004). Hypokalaemia in ibuprofen and codeine phosphate abuse. Int J Clin Pract.

[13] Chetty R, Baoku Y, Mildner R (2003). Severe hypokalaemia and weakness due to Nurofen® misuse. Ann Clin Biochem.

[14] Kane S Ibuprofen (2019). Ibuprofen. Drug usage statistics, United States, 2006 - 2016. https://clincalc.com/DrugStats/Drugs/Ibuprofen.

